# Staging of renal cell carcinoma: Current concepts

**DOI:** 10.4103/0970-1591.57906

**Published:** 2009

**Authors:** John S. Lam, Tobias Klatte, Alberto Breda

**Affiliations:** Roy and Patricia Disney Family Cancer Center, Providence Saint Joseph Medical Center, Burbank, CA 91505

**Keywords:** Kidney cancer, molecular markers, nomograms, prognosis, staging

## Abstract

The most important and widely utilized system for providing prognostic information following surgical management for renal cell carcinoma (RCC) is currently the tumor, nodes, and metastasis (TNM) staging system. An accurate and clinically useful staging system is an essential tool used to provide patients with counseling regarding prognosis, select treatment modalities, and determining eligibility for clinical trials. Data published over the last few years has led to significant controversies as to whether further revisions are needed and whether improvements can be made with the introduction of new, more accurate predictive prognostic factors. Staging systems have also evolved with an increase in the understanding of RCC tumor biology. Molecular tumor biomarkers are expected to revolutionize the staging of RCC by providing more effective prognostic ability over traditional clinical variables alone. This review will examine the components of the TNM staging system, current staging modalities including comprehensive integrated staging systems, and predictive nomograms, and introduce the concept of molecular staging for RCC.

## INTRODUCTION

Over 200,000 new cases of kidney cancer are diagnosed and more than 100,000 deaths occur from this disease each year globally.[1] Renal cell carcinoma (RCC) accounts for 3% of all adult malignancies and is steadily increasing at a rate of about 2.5% per year across population groups with the greatest increase observed in the incidence of localized tumors.[2–4] RCC is a highly aggressive tumor and the most lethal of urologic malignancies.[5] Approximately one-third of patients present with metastatic disease and up to 40% of patients will recur following surgery for clinically localized disease.[6] Significant advances in the diagnosis, staging, and treatment of patients with RCC have resulted in improved survival of a select group of patients and an overall change in the natural history of the disease.[3,7,8]

## TUMOR,  NODE,  AND  METASTASIS  (TNM) STAGING SYSTEM

Anatomical criteria have traditionally been used to stage RCC. Flocks and Kadesky[9] were the first to propose a formal staging system that stratified patients according to tumor involvement based on physical tumor characteristics and the location of tumor spread. Robson, *et al*.[10] later modified these criteria to include vascular involvement and reported an overall 52% 5-year survival and a 66% 5-year survival in patients with localized disease. This study drew attention to the basic surgical principles for the successful management of renal tumors and the importance of stratifying the anatomic spread of the tumor for the purpose of patient prognostication. These basic principles included early ligation of the renal vessels to minimize risk of vascular tumor emboli, resection of Gerota's fascia including the kidney and adrenal gland, and extensive lymphadenectomy including the para-aortic and paracaval nodes from the crus of the diaphragm to the bifurcation of the aorta. Similarly, the tumor, nodes, and metastasis (TNM) system proposed by the Union Internationale Contre le Cancer (UICC) systematically assesses local tumor growth, lymph node involvement, and distant metastasis to categorize patients.[11]

The primary size of the tumor is a key component of the TNM staging system and remains one of the most important prognostic factors for RCC.[7,8] In 1997, the cut-off size for the T1 stage was expanded from 2.5 to 7 cm, which has led to controversy.[12,13] The increasing widespread and now mainstream use of a partial nephrectomy for smaller tumors has also made the T1 cutoff criteria not only important in terms of prognostic value, but also in relation to eligibility for this surgery. Hafez, *et al*.[14] attempted to delineate the optimal cutoff size for tumors amenable to a partial nephrectomy. Patients with T1 tumors 4 cm or smaller who underwent a partial nephrectomy had a significantly better survival rate when compared with those who had larger tumors. Over the past decade, evidence from other major clinical series has shown the effectiveness and safety of partial nephrectomies in the treatment of renal tumors 4 cm or smaller.[15–19] As a result, the 2002 TNM T1 category was amended to T1a and T1b based on a 4 cm cutoff.[20] Although an elective partial nephrectomy is generally performed in patients with a tumor smaller than 4 cm, there is emerging data that it can be performed on patients with larger tumors that are anatomically amenable, provided an adequate surgical margin can be safely obtained.[21,22]

Several investigators have attempted to further improve the prognostic accuracy of T2 tumors by stratifying based on size. Frank, *et al*.[23] found that tumors larger than 10 cm behaved more aggressively compared with those between 7 and 10 cm after adjusting for regional lymph node involvement and distant metastases. In an international multicenter study, Klatte, *et al*.[24] reported that tumors larger than 11 cm were associated with the presence of metastatic disease compared with those between 7 and 11 cm. Stratification by a tumor size cut-off of 11 cm demonstrated a 5- and 10-year survival of 73% and 65% for T2 tumors 11 cm or smaller and 57% and 49% for tumors greater than 11 cm, respectively. Tumor size was retained as an independent prognostic factor for survival and was the strongest prognostic factor for patients with non-metastatic T2 disease.

A 5-year cancer-specific survival rate for T3 disease ranges from 37% to 67%, which reflects this broad category that includes various clinical situations that involve tumor extension beyond the renal capsule.[25,26] Tumors that extend into the perirenal fat but not beyond Gerota's fascia or have direct adrenal involvement are currently classified as T3a. The impact of fat invasion on the prognosis of patients with RCC is well documented. However, different locations of fat invasion have been reported to portend different prognoses. Thompson, *et al*.[27] reported that patients with renal sinus fat invasion were 1.6 times more likely to die of RCC compared with those with peripheral perinephric fat invasion. Furthermore, the risk of death persisted in multivariate analysis after adjusting for regional lymph node involvement and the presence of distant metastases. In contrast, Margulis, *et al*.[28] reported no difference in the 5-year cancer-specific survival rate between patients with sinus fat invasion and those with perinephric fat invasion only. In addition, neither sinus fat invasion nor the location of extrarenal extension correlated with the cancer-specific survival rate following surgical treatment. The presence of renal fat involvement appears to increase the risk of death from RCC among patients with venous tumor thrombus.[29–31] Leibovich, *et al*.[29] reported that among patients with T3b disease, those with concomitant perinephric or sinus fat invasion were 1.87 times more likely to die of RCC compared with patients without fat invasion. Klatte, *et al*.[30] reported that concomitant perinephric fat invasion among patients with T3b and T3c disease was an independent prognostic factor and that redefinition of the T3 classification with the incorporation of fat invasion improved prognostic accuracy.

The role of tumor size in T3a tumors has attracted little attention in literature. Siemer, *et al*.[32] analyzed patients with perinephric fat invasion and identified an ideal tumor size cut-off of 7 cm. Patients with T3a tumors 7 cm or smaller yielded similar survival to patients with T1 tumors and patients with T3a tumors larger than 7 cm yielded similar survival to T2 tumors. The current T1-2 and T3a classification for RCC has been debated in other studies. Murphy, *et al*.[33] reported worse outcomes for patients with T2 disease compared with those patients with T3a disease suggesting that tumor size was a stronger prognostic factor than tumor invasion through the renal capsule. Gilbert, *et al*.[34] also reported similar outcomes between patients with T1-2 and T3a disease. Siddiqui, *et al*.[35] investigated the association of perinephric and sinus fat invasion with death from RCC independent of tumor size. Patients with fat invasion and tumors 4 cm or smaller, 4 to 7 cm, and larger than 7 cm were 6.15, 4.13, and 2.12 times, respectively more likely to die from RCC compared with those without fat invasion. Lam, *et al*.[36] reported no difference in the cancer-specific survival rate between patients with T2 and T3a tumors 7 cm or smaller, but both groups were superior to patients with T3a tumors larger than 7 cm. Furthermore, patients with T3a tumors larger than 7 cm had a 1.36-fold increased risk of death from RCC compared with patients with T3a tumors 7 cm or smaller and T2 tumors. In addition, patients with T3a tumors larger than 7 cm had the same prognosis as patients with T3b tumors.

A few patients presented with RCC involving the ipsilateral adrenal gland at the time of diagnosis.[37,38] The current TNM staging system also categorizes patients with adrenal involvement into the T3a group. Recent reports have shown that patients with direct extension into the adrenal gland fair worse than those with extension into perirenal fat only.[39,40] Han, *et al*.[39] reported that patients with adrenal involvement faired worse than those with perinephric fat invasion and no adrenal involvement. Furthermore, the survival of patients with T3a disease and adrenal involvement was not better than patients with T4 tumors. Although a correlation existed between adrenal invasion and higher tumor grade and lymph node involvement and metastatic disease, adrenal invasion was found to be an independent predictor of poor prognosis. In addition, it has been shown that stage for stage patients with direct adrenal invasion fair worse than those without.[41] Others have corroborated the above findings concluding that tumors with adrenal involvement from direct extension appear to have a similar outcome to patients with T4 disease.[40,42] Several studies have suggested that removal of the ipsilateral adrenal gland is not routinely necessary during a radical nephrectomy.[37,43,44] Current evidence suggests that the rate of adrenal metastasis is low and that modern day imaging modalities are sensitive enough to pick up adrenal lesions.[38,44] Given the small percentage of patients with adrenal involvement and the use of detailed preoperative imaging, the vast majority of those with RCC can be spared the potential morbidity associated with an ipsilateral adrenalectomy.

RCC invades the venous system in 4–9% of newly diagnosed patients.[45,46] In 1997, inferior vena cava (IVC) tumor thrombus located above the diaphragm, previously stage T4, was changed to T3c and thrombus involvement below the diaphragm, previously staged T3c, was changed to T3b with renal vein (RV) involvement.[11] Most studies have found no difference in survival based on the level of IVC involvement[47,48] or based on the involvement of RV versus IVC.[48,49] However, it has been suggested recently that long-term survival may be better in patients with RV involvement compared with IVC involvement.[50] Recent studies have demonstrated a 5-year survival to range from 47% to 69% for patients with venous involvement and a tumor limited to the kidney.[51–55] With modern advances in surgical technique, a surgical resection can be performed with acceptable morbidity.[54,55] In select patients with metastatic disease, resection of the tumor thrombus followed by immunotherapy has been recommended.[55,56]

The overall risk of lymph node metastasis is approximately 20% and 5-year survival rates of patients with lymph node involvement ranges from 11–35%.[57–59] However, the risk of lymph node involvement varies depending on primary tumor stage and size, vascular involvement, presence of metastases, and extent of lymphadenectomy performed.[57,60] Patients with clinically localized disease have a relatively low incidence (2–9%) of nodal involvement,[60] whereas patients with metastatic disease or vascular involvement have an incidence as high as 45%.[57] In a review of 900 patients, positive lymph node status was associated with larger, higher grade, more locally advanced tumors more likely to demonstrate sarcomatoid features and were 3 to 4 times more likely to have distant metastatic disease.[57] Furthermore, patients with metastatic RCC and concomitant lymph node involvement demonstrated a significantly worse outcome compared with patients with metastatic disease alone. Patients with nodal disease manifested poorer response rates to immunotherapy.[58] However, patients with node-positive disease who underwent a lymphadenectomy had better responses to immunotherapy and higher survival rates compared with patients whose involved lymph nodes were left in place.[58] Vasselli, *et al*.[59] also reported that patients without preoperative evidence of lymph node involvement had a significantly longer survival rate than those with lymph node involvement.

Although it has been specified since the 6th edition of the TNM classification that histological examination of a regional lymphadenectomy specimens should routinely include 8 or more lymph nodes, few studies have challenged the N1-N2 subclassification. Previous studies have focused on the number of lymph nodes that were required for accurate staging as well as the utility and extent of the lymphadenectomy.[61] Terrone, *et al*.[62] reported no survival difference between N1 and N2 tumors in patients with locally advanced or metastatic disease. Among patients with positive lymph nodes, the two relevant prognostic cut-offs were 4 involved nodes and a 60% lymph node density cut-off. In addition, lymph node density was retained as an independent prognostic variable. Canfield, *et al*.[63] analyzed the prognostic significance of nodal disease in the absence of distant metastatic disease. The median survival rate in patients with N2 disease was significantly worse compared with patients with N1 disease. In addition, more than 1 positive node was an independent predictor of decreased disease-free and overall survival. Dimashkieh, *et al*.[64] examined the associations of pathological features of lymph node metastases with outcome in a cohort of patients treated with radical nephrectomy for unilateral, sporadic M0 RCC. There was no significant difference in the survival rate between patients with N1 and N2 disease. However, patients with extranodal extension were twice as likely to die of RCC compared with patients in whom metastases did not extend outside of the lymph node capsule.

## COMPREHENSIVE INTEGRATED STAGING SYSTEMS AND PREDICTIVE NOMOGRAMS

The anatomical, histological, and clinical factors that influence disease recurrence and survival in RCC make counseling patients particularly challenging. Many centers have aimed to integrate these independent prognostic indicators into comprehensive outcome models for both non metastatic and metastatic RCC to assist clinicians in facilitating patient counseling and identifying those patients who might benefit from treatment. The first report addressing this issue appeared in 1986 in which the factors predicting outcome for patients with metastatic RCC included performance status (PS), presence of pulmonary metastases, and metastatic-free interval.[65] More recently, several groups have created similar models designed to include patients with localized and metastatic disease.

Elson, *et al*.[66] presented an analysis of 610 patients with recurrent or metastatic RCC who had been treated with chemotherapy in clinical trials sponsored by the Eastern Cooperative Oncology Group (ECOG). A scoring system was developed to stratify patients into 5 categories based on PS (0 to 1 *vs*. 2 to 3), time from initial diagnosis (>1 year *vs*. 1 year), number of metastatic sites, prior cytotoxic chemotherapy, and recent weight loss. The Karnofsky or ECOG-PS scales are a convenient common denominator for the overall impact of multiple objective and subjective symptoms and signs on patients. Using this system, median survival times ranging from 2.1 to 12.8 months were observed across the five separate categories. As this cohort was examined prior to the initiation of immunotherapy, its validity for today's patient population is questionable.

Motzer, *et al*.[67] developed a model based on 670 patients with advanced RCC treated in 24 separate clinical trials at the Memorial Sloan-Kettering Cancer Center (MSKCC) including 394 patients treated with IFN-α or IL-2. This model was created by defining the relationship of pretreatment clinical features and survival, which included the following risk factors: low Karnofsky PS score, high serum lactate dehydrogenase levels, low hemoglobin levels, hypercalcemia, and prior nephrectomy. The median survival rate was 10 months and significantly shorter survival occurred in patients with poor PS (Karnofsky scale <80%), high lactate dehydrogenase (>1.5 times the upper limit of normal), low hemoglobin, high-corrected calcium (>10 mg/dL), and absence of prior nephrectomy. Patients were stratified into favorable-, intermediate-, and poor- risk groups according to the number of risk factors present. Patients at poor risk with 3 or more risk factors had a median survival of 4 months, whereas median survival improved to 20 months in those with no risk factors.

To analyze prognostic factors that would benefit modern day clinical trials, Motzer, *et al*.[68] reviewed 137 patients with metastatic RCC enrolled in clinical trials at MSKCC from 1990 onwards. The median overall survival rate for this group was 12.7 months. Independent predictors of worse survival rates were poor PS (Karnofsky scale <80%), low hemoglobin levels (less than or equal to 13 g/dL in males and 11.5 g/dL in females), and elevated corrected serum calcium (≥10 mg/dL). The number of poor prognostic variables stratified patients into favorable-risk (no risk factors), intermediate-risk (one risk factor), and poor-risk (two or three risk factors) groups. The favorable-, intermediate-, and poor-risk groups demonstrated overall 1- and 3-year survival rates of 76% and 25%, 49% and 11%, and 11% and 0%, respectively.

A study of 353 patients with previously untreated advanced RCC at the Cleveland Clinic was conducted to assess and validate the model proposed from MSKCC.[69] Four of five prognostic factors (time from diagnosis to entry into the study, serum lactate dehydrogenase, corrected serum calcium, and hemoglobin) identified by the MSKCC group were independent predictors of survival. In addition, prior radiotherapy and the presence of hepatic, lung, and retroperitoneal nodal metastases were found to be independent prognostic factors. Using the number of metastatic sites as surrogates for individual sites (none or one *vs*. two or three sites), the MSKCC definitions of risk groups were expanded to accommodate these two additional prognostic factors. Using this expanded criteria, favorable-risk was defined as zero or one poor prognostic factor, intermediate-risk was defined as two poor prognostic factors, and poor-risk was defined as more than two poor prognostic factors.

The International Kidney Cancer Working Group is currently establishing a comprehensive database from centers that treat patients with metastatic RCC. This will be used to develop a set of prognostic factors in patients with metastatic RCC and ultimately to derive a single validated model. Preliminary studies were performed to determine the availability of a database that could be used for the planned analysis of prognostic factors, which involved the examination of 782 patients treated by the Groupe Francais d'Immunotherapie[70] and patients treated at the Cleveland Clinic.[69] These two groups were similar in their distribution of various clinical factors and survival.[71] These findings suggest that the use of an international database would be a reasonable approach to identify prognostic factors and validate a model for patients with this disease. Additionally, this database could serve as a resource to study the natural history of this illness and aid in the design and analysis of clinical trials for patients with metastatic RCC.

The Kattan postoperative prognostic nomogram[72] was created to predict the probability of tumor recurrence within 5 years in patients undergoing a radical nephrectomy for RCC. The nomogram assigns numerical scores to various prognostic indicators that include presence of symptoms, histology, tumor size, and TNM staging criteria. In a study of 601 patients with RCC who were treated with a nephrectomy, the nomogram appeared accurate and discriminating with an area under the receiver operating curve of 0.74. This nomogram was later modified to exclude histologic subtype and the analysis was limited to clear cell RCC.[73] The 5-year probability of freedom from recurrence for the patient cohort was 80.9%. This nomogram has a concordance index of 0.82 and external validation revealed it to be accurate and discriminating.

The UCLA integrated staging system (UISS) is an extensive prognostic system that has been created for both localized and metastatic RCC.[74] The initial UISS contained 5 groups based on TNM stage, Fuhrman grade, and ECOG-PS all found to be independent predictors of survival. Projected 2-year survival rates and 5-year survival rates for patients in UISS Groups I through V were 96% and 94%, 89% and 67%, 66% and 39%, 42% and 23%, and 9% and 0%, respectively. The UISS was internally validated using a bootstrapping technique and an expanded database of patients treated at UCLA between 1989 and 2000,[75] external data from patients treated at MD Anderson Cancer Center, patients treated in Nijmegen, Netherlands,[76] and most recently with 4,202 patients from 8 international centers.[77] The UISS has been subsequently modified into a simplified system based on separate stratification of metastatic and non metastatic patients into low-, intermediate-, and high-risk groups.[78] This provides a clinically useful system for predicting postoperative outcome and provides a unique tool for risk assignment and outcome analysis to help determine follow-up regimens and eligibility for clinical trials.

The Mayo Clinic created an extensive outcome prediction model for patients with clear cell RCC who are undergoing a radical nephrectomy.[79] According to an analysis of data from 1,801 patients, TNM stage, tumor size smaller than 5 cm, nuclear grade, and the presence of histological tumor necrosis were all found to be independent predictors of survival. Histologic necrosis is defined as any degree of microscopic tumor necrosis exclusive of degenerative changes such as hyalinization, hemorrhage, or fibrosis. The presence of tumor necrosis has been recognized to be associated with markers of advanced disease,[79–81] as well as an independent predictor of survival.[79,81,82] These factors were combined into the stage, size, grade, and necrosis (SSIGN) scoring algorithm. Decreased survival was shown to correlate with an increased SSIGN score with scores of 0–1 and greater than 10 correlating with a 5-year cancer-specific survival rate of 99% and 7%, respectively.

In the metastatic setting, it is well accepted that PS is a strong predictor for survival. Similarly, several studies have shown that cancer-related symptoms were independent prognostic parameters in localized RCC.[83–85] Recently, a symptom-based classification was established and externally validated in a large multicenter series.[86,87] Based on symptoms at diagnosis, 388 renal tumors were stratified into three groups: asymptomatic tumors (S1), tumors with isolated local symptoms (S2), and tumors with systemic symptoms (S3). The S classification appeared to predict cancer-specific survival independent of tumor stage and grade. Two different large subsets of patients that integrated both tumor size and tumor-related symptom information within the TNM classification have resulted in improved prognostic stratification.[88,89] Karakiewicz, *et al*.[90] recently proposed a highly accurate (86.3%) prognostic nomogram that consisted of TNM stage, Fuhrman grade, tumor size, and symptom classification. External validation of the nomogram at 1, 2, 5, and 10 years after a nephrectomy revealed predictive accuracy of 87.8%, 89.2%, 86.7%, and 88.8%, respectively. Conversely, the UISS, which predicts cancer-specific survival rates at 2 and 5 years, was less accurate as evidenced by 86.1% and 83.9% estimates. Furthermore, the Karakiewicz model provides accurate predictions that span a 10-year period after a nephrectomy, which exceeds the prognostic range of other models.

The presence of distant metastases at diagnosis substantially changes the prognosis of patients with RCC.[91,92] Leibovich, *et al*.[93] reported that in patients with non metastatic clear cell RCC who were treated with a radical nephrectomy, the median time to distant metastases was 1.3 years and of those who progressed to distant metastases, 77% died from RCC. Hutterer, *et al*.[94] recently developed a nomogram based on symptom classification and tumor size derived from a multi-institutional database aimed at quantifying the risk of distant metastases in patients with RCC. This nomogram was 85.2% accurate in predicting the individual probability of distant metastases in an external validation cohort and may assist in identifying patients at high risk of having metastatic RCC.

The outcome of patients with RCC nodal metastases is substantially worse than that of patients with localized disease. Hutterer, *et al*.[95] developed and externally validated a nomogram based on age, symptom classification, and tumor size that is accurate (78.4%) in predicting RCC nodal metastases in patients without radiographic evidence of distant metastases. This tool can help to risk adjust the need and the extent of nodal staging in patients without known distant metastases. More thorough staging can hopefully better select those in whom adjuvant treatment is necessary. As of today there are no clear guidelines regarding exactly who should be subjected to a staging lymphadenectomy with the intent of identifying those with nodal metastases. For example, Blom, *et al*.[96] demonstrated that in non metastatic T1-3 RCC patients, the rate of unsuspected lymph node metastases is as low as 3.3%. Such data indicate that the majority of RCC patients will be free of nodal metastases.

## MOLECULAR STAGING

Molecular biomarkers may prove more effective for predicting survival than traditional clinical parameters such as tumor stage and grade.[97] The next generation of prognostic models hopes to incorporate the advancements of molecular biology and genetics. Methods based on gene arrays, which screen for differential expression of thousands of genes, have identified large numbers of new, potentially important prognostic markers in RCC.[98–101] A useful tool for validating a limited number of biomarkers on a large patient population is the tissue microarray (TMA). Sections of the TMA provide targets for parallel *in situ* detection of DNA, RNA, and protein in the same set of specimens, which can be correlated to clinical data with respect to disease progression, treatment response, and survival. The evaluation of protein expression in a high-throughput TMA is a natural extension to the efforts for molecular staging. Accurate models for predicting survival can be constructed using multiple molecular biomarkers. Kim, *et al*.[102,103] have recently demonstrated that molecular characterization improves upon the UISS. Immunohistochemical analysis of 8 molecular biomarkers previously linked to the development of malignancies (Ki-67, p53, gelsolin, carbonic anhydrase [CA] IX, CA XII, PTEN [phosphatase and tensin homologue deleted on chromosome 10], epithelial cell adhesion molecule [EpCAM], and vimentin) was performed on a custom TMA using clear cell RCC from 318 patients, representing all stages of localized and metastatic RCC. A prognostic model based primarily on molecular markers included metastasis status, p53, CA IX, gelsolin, and vimentin as predictors and had high discriminatory power; its statistically validated concordance index (C-index) was found to be 0.75. A prognostic model based on a combination of clinical and molecular predictors included metastasis status, T stage, ECOG-PS, p53, CA IX, and vimentin as predictors and had a C-index of 0.79, which was significantly higher than that of prognostic models based on grade alone (C = 0.65), TNM stage alone (C = 0.73), or the UISS (C = 0.76).

Two nomograms were proposed that could be used to predict disease-specific survival. One nomogram is based on metastasis status and molecular markers (CA IX, p53, vimentin, and gelsolin). The second nomogram combines clinical and molecular variables (metastasis status, T-stage, ECOG-PS, CA IX, p53, and vimentin). By including metastasis status, the nomograms accurately predict cancer-specific survival in patients with both localized and metastatic RCC. Both nomograms can be used to calculate 2- and 4-year cancer-specific survival rates as well as median survival. This study shows that accurate models for molecular staging of a solid tumor can be developed using a very limited number of markers. Although these nomograms are useful for visualizing our predictive models, they need to be validated on independent patient populations prior to being applied to patient care.

Gene expression analysis studies have also demonstrated the ability to define patient prognosis. Takahashi, *et al*.[104] showed that clear cell RCC tumors exhibited a high expression of VEGF and ceruloplasmin and down-regulation of kininogen. The authors identified 40 genes that identified patients with the best prognosis. Increased expression of SPROUTY, an angiogenesis inhibitor, was associated with a good prognosis, while loss of transforming growth factor beta receptor II and MMP-3 were associated with poor outcomes. Takashashi, *et al*.[105] also identified a unique gene expression profile in clear cell RCC that differentiated between patients who died from their disease and those with no evidence of metastasis. Jones, *et al*.[106] reported the ability of gene expression analysis within the primary tumor to identify a metastatic signature, which includes topoisomerase IIα and glycosyl ceramide synthase. Vasselli, *et al*.[98] identified 45 genes to separate patients with metastatic RCC into groups based on prognosis. Increased expression of vascular cell adhesion molecule-1 was found to be particularly powerful in selecting patients with improved prognosis.

## CONCLUSIONS

Over the last 10 years, there has been a gradual transition from the use of solitary clinical factors as prognostic markers for patients with RCC to the introduction of systems that integrate multiple factors to the introduction of molecular and genetic markers with the goal of improving patient prognostication. The field of RCC is rapidly undergoing a revolution led by molecular biomarkers. The understanding of tumor biology gleaned from molecular biomarker research will be critical to the future treatment of patients with RCC.

## References

[1] Parkin DM, Bray F, Ferlay J, Pisani P (2005). Global cancer statistics, 2002. CA Cancer J Clin.

[2] Chow WH, Devesa SS, Warren JL, Fraumeni JF (1999). Rising incidence of renal cell cancer in the United States. JAMA.

[3] Pantuck AJ, Zisman A, Belldegrun AS (2001). The changing natural history of renal cell carcinoma. J Urol.

[4] Hock LM, Lynch J, Balaji KC (2002). Increasing incidence of all stages of kidney cancer in the last 2 decades in the United States: an analysis of surveillance, epidemiology and end results program data. J Urol.

[5] Jemal A, Siegel R, Ward E, Murray T, Xu J, Thun MJ (2007). Cancer statistics, 2007. CA Cancer J Clin.

[6] Lam JS, Leppert JT, Figlin RA, Belldegrun AS (2005). Surveillance following radical or partial nephrectomy for renal cell carcinoma. Curr Urol Rep.

[7] Lam JS, Shvarts O, Leppert JT, Figlin RA, Belldegrun AS (2005). Renal cell carcinoma 2005: new frontiers in staging, prognostication and targeted molecular therapy. J Urol.

[8] Lam JS, Breda A, Belldegrun AS, Figlin RA (2006). Evolving principles of surgical management and prognostic factors for outcome in renal cell carcinoma. J Clin Oncol.

[9] Flocks RH, Kadesky MC (1958). Malignant neoplasms of the kidney; an analysis of 353 patients followed five years or more. J Urol.

[10] Robson CJ, Churchill BM, Anderson W (1969). The results of radical nephrectomy for renal cell carcinoma. J Urol.

[11] Guinan P, Sobin LH, Algaba F, Badellino F, Kameyama S, MacLennan G (1997). TNM staging of renal cell carcinoma: Workgroup No. 3. Union International Contre le Cancer (UICC) and the American Joint Committee on Cancer (AJCC). Cancer.

[12] Guinan P, Saffrin R, Stuhldreher D, Frank W, Rubenstein M (1995). Renal cell carcinoma: comparison of the TNM and Robson stage groupings. J Surg Oncol.

[13] Hermanek P, Schrott KM (1990). Evaluation of the new tumor, nodes and metastases classification of renal cell carcinoma. J Urol.

[14] Hafez KS, Novick AC, Campbell SC (1997). Patterns of tumor recurrence and guidelines for followup after nephron sparing surgery for sporadic renal cell carcinoma. J Urol.

[15] Lerner SE, Hawkins CA, Blute ML, Grabner A, Wollan PC, Eickholt JT (1996). Disease outcome in patients with low stage renal cell carcinoma treated with nephron sparing or radical surgery. J Urol.

[16] Belldegrun A, Tsui KH, deKernion JB, Smith RB (1999). Efficacy of nephron-sparing surgery for renal cell carcinoma: analysis based on the new 1997 tumor-node-metastasis staging system. J Clin Oncol.

[17] Lee CT, Katz J, Shi W, Thaler HT, Reuter VE, Russo P (2000). Surgical management of renal tumors 4 cm. or less in a contemporary cohort. J Urol.

[18] Fergany AF, Hafez KS, Novick AC (2000). Long-term results of nephron sparing surgery for localized renal cell carcinoma: 10-year followup. J Urol.

[19] Becker F, Siemer S, Humke U, Hack M, Ziegler M, Stockle M (2005). Elective Nephron Sparing Surgery Should Become Standard Treatment for Small Unilateral Renal Cell Carcinoma: Long-term Survival Data of 216 Patients. Eur Urol.

[20] Sobin LH, Wittekind C (2002). TNM Classification of Malignant Tumours.

[21] Leibovich BC, Blute ML, Cheville JC, Lohse CM, Weaver AL, Zincke H (2004). Nephron sparing surgery for appropriately selected renal cell carcinoma between 4 and 7 cm results in outcome similar to radical nephrectomy. J Urol.

[22] Patard JJ, Shvarts O, Lam JS, Pantuck AJ, Kim HL, Ficarra V (2004). Safety and efficacy of partial nephrectomy for all T1 tumors based on an international multicenter experience. J Urol.

[23] Frank I, Blute ML, Leibovich BC, Cheville JC, Lohse CM, Kwon ED (2005). pT2 classification for renal cell carcinoma. Can its accuracy be improved?. J Urol.

[24] Klatte T, Patard JJ, Goel RH, Kleid MD, Guille F, Lobel B (2007). Prognostic Impact of Tumor Size on pT2 Renal Cell Carcinoma: An International Multicenter Experience. J Urol.

[25] Tsui KH, Shvarts O, Smith RB, Figlin RA, deKernion JB, Belldegrun A (2000). Prognostic indicators for renal cell carcinoma: a multivariate analysis of 643 patients using the revised 1997 TNM staging criteria. J Urol.

[26] Frank I, Blute ML, Leibovich BC, Cheville JC, Lohse CM, Zincke H (2005). Independent validation of the 2002 American Joint Committee on cancer primary tumor classification for renal cell carcinoma using a large, single institution cohort. J Urol.

[27] Thompson RH, Leibovich BC, Cheville JC, Webster WS, Lohse CM, Kwon ED (2005). Is renal sinus fat invasion the same as perinephric fat invasion for pT3a renal cell carcinoma?. J Urol.

[28] Margulis V, Tamboli P, Matin SF, Meisner M, Swanson DA, Wood CG (2007). Location of extrarenal tumor extension does not impact survival of patients with pT3a renal cell carcinoma. J Urol.

[29] Leibovich BC, Cheville JC, Lohse CM, Zincke H, Kwon ED, Frank I (2005). Cancer specific survival for patients with pT3 renal cell carcinoma-can the 2002 primary tumor classification be improved?. J Urol.

[30] Klatte T, Pantuck AJ, Riggs SB, Kleid MD, Shuch B, Zomorodian N (2007). Prognostic factors for renal cell carcinoma with tumor thrombus extension. J Urol.

[31] Margulis V, Tamboli P, Matin SF, Meisner M, Swanson DA, Wood CG (2007). Redefining pT3 renal cell carcinoma in the modern era: a proposal for a revision of the current TNM primary tumor classification system. Cancer.

[32] Siemer S, Lehmann J, Loch A, Becker F, Stein U, Schneider G (2005). Current TNM classification of renal cell carcinoma evaluated: revising stage T3a. J Urol.

[33] Murphy AM, Gilbert SM, Katz AE, Goluboff ET, Sawczuk IS, Olsson CA (2005). Re-evaluation of the Tumour-Node-Metastasis staging of locally advanced renal cortical tumours: absolute size (T2) is more significant than renal capsular invasion (T3a). BJU Int.

[34] Gilbert SM, Murphy AM, Katz AE, Goluboff ET, Sawczuk IS, Olsson CA (2006). Reevaluation of TNM staging of renal cortical tumors: recurrence and survival for T1N0M0 and T3aN0M0 tumors are equivalent. Urology.

[35] Siddiqui SA, Frank I, Leibovich BC, Cheville JC, Lohse CM, Zincke H (2007). Impact of tumor size on the predictive ability of the pT3a primary tumor classification for renal cell carcinoma. J Urol.

[36] Lam JS, Klatte T, Patard JJ, Goel RH, Guille F, Lobel B (2007). Prognostic relevance of tumour size in t3a renal cell carcinoma: a multicentre experience. Eur Urol.

[37] Kletscher BA, Qian J, Bostwick DG, Blute ML, Zincke H (1996). Prospective analysis of the incidence of ipsilateral adrenal metastasis in localized renal cell carcinoma. J Urol.

[38] Tsui KH, Shvarts O, Barbaric Z, Figlin R, de Kernion JB, Belldegrun A (2000). Is adrenalectomy a necessary component of radical nephrectomy? UCLA experience with radical nephrectomies. J Urol.

[39] Han KR, Bui MH, Pantuck AJ, Freitas DG, Leibovich BC, Dorey FJ (2003). TNM T3a renal cell carcinoma: adrenal gland involvement is not the same as renal fat invasion. J Urol.

[40] Thompson RH, Leibovich BC, Cheville JC, Lohse CM, Frank I, Kwon ED (2005). Should direct ipsilateral adrenal invasion from renal cell carcinoma be classified as pT3a?. J Urol.

[41] Thompson RH, Cheville JC, Lohse CM, Webster WS, Zincke H, Kwon ED (2005). Reclassification of patients with pT3 and pT4 renal cell carcinoma improves prognostic accuracy. Cancer.

[42] Ficarra V, Novara G, Iafrate M, Cappellaro L, Bratti E, Zattoni F (2007). Proposal for Reclassification of the TNM Staging System in Patients with Locally Advanced (pT3-4) Renal Cell Carcinoma According to the Cancer-Related Outcome. Eur Urol.

[43] Paul R, Mordhorst J, Busch R, Leyh H, Hartung R (2001). Adrenal sparing surgery during radical nephrectomy in patients with renal cell cancer: a new algorithm. J Urol.

[44] Siemer S, Lehmann J, Kamradt J, Loch T, Remberger K, Humke U (2004). Adrenal metastases in 1635 patients with renal cell carcinoma: outcome and indication for adrenalectomy. J Urol.

[45] Pagano F, Dal Bianco M, Artibani W, Pappagallo G, Prayer Galetti T (1992). Renal cell carcinoma with extension into the inferior vena cava: problems in diagnosis, staging and treatment. Eur Urol.

[46] Hatcher PA, Anderson EE, Paulson DF, Carson CC, Robertson JE (1991). Surgical management and prognosis of renal cell carcinoma invading the vena cava. J Urol.

[47] Sosa RE, Muecke EC, Vaughan ED, McCarron JP (1984). Renal cell carcinoma extending into the inferior vena cava: the prognostic significance of the level of vena caval involvement. J Urol.

[48] Kim HL, Zisman A, Han KR, Figlin RA, Belldegrun AS (2004). Prognostic significance of venous thrombus in renal cell carcinoma. Are renal vein and inferior vena cava involvement different?. J Urol.

[49] Ljungberg B, Stenling R, Osterdahl B, Farrelly E, Aberg T, Roos G (1995). Vein invasion in renal cell carcinoma: impact on metastatic behavior and survival. J Urol.

[50] Moinzadeh A, Libertino JA (2004). Prognostic significance of tumor thrombus level in patients with renal cell carcinoma and venous tumor thrombus extension. Is all T3b the same?. J Urol.

[51] Skinner DG, Pritchett TR, Lieskovsky G, Boyd SD, Stiles QR (1989). Vena caval involvement by renal cell carcinoma. Surgical resection provides meaningful long-term survival. Ann Surg.

[52] Libertino JA, Zinman L, Watkins E (1987). Long-term results of resection of renal cell cancer with extension into inferior vena cava. J Urol.

[53] Glazer AA, Novick AC (1996). Long-term followup after surgical treatment for renal cell carcinoma extending into the right atrium. J Urol.

[54] Zisman A, Wieder JA, Pantuck AJ, Chao DH, Dorey F, Said JW (2003). Renal cell carcinoma with tumor thrombus extension: biology, role of nephrectomy and response to immunotherapy. J Urol.

[55] Parekh DJ, Cookson MS, Chapman W, Harrell F, Wells N, Chang SS (2005). Renal cell carcinoma with renal vein and inferior vena caval involvement: clinicopathological features, surgical techniques and outcomes. J Urol.

[56] Naitoh J, Kaplan A, Dorey F, Figlin R, Belldegrun A (1999). Metastatic renal cell carcinoma with concurrent inferior vena caval invasion: long-term survival after combination therapy with radical nephrectomy, vena caval thrombectomy and postoperative immunotherapy. J Urol.

[57] Pantuck AJ, Zisman A, Dorey F, Chao DH, Han KR, Said J (2003). Renal cell carcinoma with retroperitoneal lymph nodes: role of lymph node dissection. J Urol.

[58] Pantuck AJ, Zisman A, Dorey F, Chao DH, Han KR, Said J (2003). Renal cell carcinoma with retroperitoneal lymph nodes. Impact on survival and benefits of immunotherapy. Cancer.

[59] Vasselli JR, Yang JC, Linehan WM, White DE, Rosenberg SA, Walther MM (2001). Lack of retroperitoneal lymphadenopathy predicts survival of patients with metastatic renal cell carcinoma. J Urol.

[60] Terrone C, Guercio S, De Luca S, Poggio M, Castelli E, Scoffone C (2003). The number of lymph nodes examined and staging accuracy in renal cell carcinoma. BJU Int.

[61] Joslyn SA, Sirintrapun SJ, Konety BR (2005). Impact of lymphadenectomy and nodal burden in renal cell carcinoma: retrospective analysis of the National Surveillance, Epidemiology, and End Results database. Urology.

[62] Terrone C, Cracco C, Porpiglia F, Bollito E, Scoffone C, Poggio M (2006). Reassessing the current TNM lymph node staging for renal cell carcinoma. Eur Urol.

[63] Canfield SE, Kamat AM, Sanchez-Ortiz RF, Detry M, Swanson DA, Wood CG (2006). Renal cell carcinoma with nodal metastases in the absence of distant metastatic disease (clinical stage TxN1-2M0): the impact of aggressive surgical resection on patient outcome. J Urol.

[64] Dimashkieh HH, Lohse CM, Blute ML, Kwon ED, Leibovich BC, Cheville JC (2006). Extranodal extension in regional lymph nodes is associated with outcome in patients with renal cell carcinoma. J Urol.

[65] Maldazys JD, deKernion JB (1986). Prognostic factors in metastatic renal carcinoma. J Urol.

[66] Elson PJ, Witte RS, Trump DL (1988). Prognostic factors for survival in patients with recurrent or metastatic renal cell carcinoma. Cancer Res.

[67] Motzer RJ, Mazumdar M, Bacik J, Berg W, Amsterdam A, Ferrara J (1999). Survival and prognostic stratification of 670 patients with advanced renal cell carcinoma. J Clin Oncol.

[68] Motzer RJ, Bacik J, Schwartz LH, Reuter V, Russo P, Marion S (2004). Prognostic factors for survival in previously treated patients with metastatic renal cell carcinoma. J Clin Oncol.

[69] Mekhail TM, Abou-Jawde RM, Boumerhi G, Malhi S, Wood L, Elson P (2005). Validation and extension of the Memorial Sloan-Kettering prognostic factors model for survival in patients with previously untreated metastatic renal cell carcinoma. J Clin Oncol.

[70] Negrier S, Escudier B, Gomez F, Douillard JY, Ravaud A, Chevreau C (2002). Prognostic factors of survival and rapid progression in 782 patients with metastatic renal carcinomas treated by cytokines: a report from the Groupe Francais d'Immunotherapie. Ann Oncol.

[71] Bukowski RM, Negrier S, Elson P (2004). Prognostic factors in patients with advanced renal cell carcinoma: development of an international kidney cancer working group. Clin Cancer Res.

[72] Kattan MW, Reuter V, Motzer RJ, Katz J, Russo P (2001). A postoperative prognostic nomogram for renal cell carcinoma. J Urol.

[73] Sorbellini M, Kattan MW, Snyder ME, Reuter V, Motzer R, Goetzl M (2005). A postoperative prognostic nomogram predicting recurrence for patients with conventional clear cell renal cell carcinoma. J Urol.

[74] Zisman A, Pantuck AJ, Dorey F, Said JW, Shvarts O, Quintana D (2001). Improved prognostication of renal cell carcinoma using an integrated staging system. J Clin Oncol.

[75] Zisman A, Pantuck AJ, Figlin RA, Belldegrun AS (2001). Validation of the ucla integrated staging system for patients with renal cell carcinoma. J Clin Oncol.

[76] Han KR, Bleumer I, Pantuck AJ, Kim HL, Dorey FJ, Janzen NK (2003). Validation of an integrated staging system toward improved prognostication of patients with localized renal cell carcinoma in an international population. J Urol.

[77] Patard JJ, Kim HL, Lam JS, Dorey FJ, Pantuck AJ, Zisman A (2004). Use of the university of california los angeles integrated staging system to predict survival in renal cell carcinoma: an international multicenter study. J Clin Oncol.

[78] Zisman A, Pantuck AJ, Wieder J, Chao DH, Dorey F, Said JW (2002). Risk group assessment and clinical outcome algorithm to predict the natural history of patients with surgically resected renal cell carcinoma. J Clin Oncol.

[79] Frank I, Blute ML, Cheville JC, Lohse CM, Weaver AL, Zincke H (2002). An outcome prediction model for patients with clear cell renal cell carcinoma treated with radical nephrectomy based on tumor stage, size, grade and necrosis: the SSIGN score. J Urol.

[80] Amin MB, Tamboli P, Javidan J, Stricker H, de-Peralta Venturina M, Deshpande A (2002). Prognostic impact of histologic subtyping of adult renal epithelial neoplasms: an experience of 405 cases. Am J Surg Pathol.

[81] Lam JS, Shvarts O, Said JW, Pantuck AJ, Seligson DB, Aldridge ME (2005). Clinicopathologic and molecular correlations of necrosis in the primary tumor of patients with renal cell carcinoma. Cancer.

[82] Sengupta S, Lohse CM, Leibovich BC, Frank I, Thompson RH, Webster WS (2005). Histologic coagulative tumor necrosis as a prognostic indicator of renal cell carcinoma aggressiveness. Cancer.

[83] Patard JJ, Rodriguez A, Rioux-Leclercq N, Guille F, Lobel B (2002). Prognostic significance of the mode of detection in renal tumours. BJU Int.

[84] Kim HL, Belldegrun AS, Freitas DG, Bui MH, Han KR, Dorey FJ (2003). Paraneoplastic signs and symptoms of renal cell carcinoma: implications for prognosis. J Urol.

[85] Kim HL, Han KR, Zisman A, Figlin RA, Belldegrun AS (2004). Cachexia-like symptoms predict a worse prognosis in localized t1 renal cell carcinoma. J Urol.

[86] Patard JJ, Leray E, Rodriguez A, Rioux-Leclercq N, Guille F, Lobel B (2003). Correlation between symptom graduation, tumor characteristics and survival in renal cell carcinoma. Eur Urol.

[87] Patard JJ, Leray E, Cindolo L, Ficarra V, Rodriguez A, De La Taille A (2004). Multi-institutional validation of a symptom based classification for renal cell carcinoma. J Urol.

[88] Patard JJ, Dorey FJ, Cindolo L, Ficarra V, De La Taille A, Tostain J (2004). Symptoms as Well as Tumor Size Provide Prognostic Information on Patients with Localized Renal Tumors. J Urol.

[89] Ficarra V, Guille F, Schips L, de la Taille A, Prayer Galetti T, Tostain J (2005). Proposal for revision of the TNM classification system for renal cell carcinoma. Cancer.

[90] Karakiewicz PI, Briganti A, Chun FK, Trinh QD, Perrotte P, Ficarra V (2007). Multi-institutional validation of a new renal cancer-specific survival nomogram. J Clin Oncol.

[91] Parton M, Gore M, Eisen T (2006). Role of cytokine therapy in 2006 and beyond for metastatic renal cell cancer. J Clin Oncol.

[92] Karakiewicz PI, Trinh QD, Bhojani N, Bensalah K, Salomon L, de la Taille A (2007). Renal cell carcinoma with nodal metastases in the absence of distant metastatic disease: prognostic indicators and disease-specific survival. Eur Urol.

[93] Leibovich BC, Blute ML, Cheville JC, Lohse CM, Frank I, Kwon ED (2003). Prediction of progression after radical nephrectomy for patients with clear cell renal cell carcinoma: a stratification tool for prospective clinical trials. Cancer.

[94] Hutterer GC, Patard JJ, Jeldres C, Perrotte P, de La Taille A, Salomon L (2008). Patients with distant metastases from renal cell carcinoma can be accurately identified: external validation of a new nomogram. BJU Int.

[95] Hutterer GC, Patard JJ, Perrotte P, Ionescu C, de La Taille A, Salomon L (2007). Patients with renal cell carcinoma nodal metastases can be accurately identified: External validation of a new nomogram. Int J Cancer.

[96] Blom JH, van Poppel H, Marechal JM, Jacqmin D, Sylvester R, Schroder FH (1999). Radical nephrectomy with and without lymph node dissection: preliminary results of the EORTC randomized phase III protocol 30881. EORTC Genitourinary Group. Eur Urol.

[97] Lam JS, Leppert JT, Figlin RA, Belldegrun AS (2005). Role of molecular markers in the diagnosis and therapy of renal cell carcinoma. Urology.

[98] Vasselli JR, Shih JH, Iyengar SR, Maranchie J, Riss J, Worrell R (2003). Predicting survival in patients with metastatic kidney cancer by gene-expression profiling in the primary tumor. Proc Natl Acad Sci U S A.

[99] Tan MH, Rogers CG, Cooper JT, Ditlev JA, Maatman TJ, Yang X (2004). Gene expression profiling of renal cell carcinoma. Clin Cancer Res.

[100] Yang XJ, Tan MH, Kim HL, Ditlev JA, Betten MW, Png CE (2005). A molecular classification of papillary renal cell carcinoma. Cancer Res.

[101] Yang XJ, Sugimura J, Schafernak KT, Tretiakova MS, Han M, Vogelzang NJ (2006). Classification of renal neoplasms based on molecular signatures. J Urol.

[102] Kim HL, Seligson D, Liu X, Janzen N, Bui MH, Yu H (2004). Using protein expressions to predict survival in clear cell renal carcinoma. Clin Cancer Res.

[103] Kim HL, Seligson D, Liu X, Janzen N, Bui MH, Yu H (2005). Using tumor markers to predict the survival of patients with metastatic renal cell carcinoma. J Urol.

[104] Takahashi M, Rhodes DR, Furge KA, Kanayama H, Kagawa S, Haab BB (2001). Gene expression profiling of clear cell renal cell carcinoma: gene identification and prognostic classification. Proc Natl Acad Sci U S A.

[105] Takahashi M, Yang XJ, Sugimura J, Backdahl J, Tretiakova M, Qian CN (2003). Molecular subclassification of kidney tumors and the discovery of new diagnostic markers. Oncogene.

[106] Jones J, Otu H, Spentzos D, Kolia S, Inan M, Beecken WD (2005). Gene signatures of progression and metastasis in renal cell cancer. Clin Cancer Res.

